# Aminoimidazole Carboxamide Ribonucleotide (AICAR) Inhibits the Growth of Retinoblastoma In Vivo by Decreasing Angiogenesis and Inducing Apoptosis

**DOI:** 10.1371/journal.pone.0052852

**Published:** 2013-01-03

**Authors:** Sofia Theodoropoulou, Katarzyna Brodowska, Maki Kayama, Yuki Morizane, Joan W. Miller, Evangelos S. Gragoudas, Demetrios G. Vavvas

**Affiliations:** Angiogenesis Laboratory, Massachusetts Eye and Ear Infirmary, Department of Ophthalmology, Retina Service, Harvard Medical School, Boston, Massachusetts, United States of America; The University of Kansas Medical Center, United States of America

## Abstract

5-Aminoimidazole-4-carboxamide-1-β-4-ribofuranoside (AICAR), an analog of AMP is widely used as an activator of AMP-kinase (AMPK), a protein that regulates the responses of the cell to energy change. Recently, we showed that AICAR-induced AMPK activation inhibits the growth of retinoblastoma cells *in vitro* by decreasing cyclins and by inducing apoptosis and S-phase arrest. In this study, we investigated the effects of AMPK activator AICAR on the growth of retinoblastoma *in vivo*. Intraperitoneal injection of AICAR resulted in 48% growth inhibition of Y79 retinoblastoma cell tumors in mice. Tumors isolated from mice treated with AICAR had decreased expression of Ki67 and increased apoptotic cells (TUNEL positive) compared with the control. In addition, AICAR treatment suppressed significantly tumor vessel density and macrophage infiltration. We also showed that AICAR administration resulted in AMPK activation and mTOR pathway inhibition. Paradoxically observed down-regulation of p21, which indicates that p21 may have a novel function of an oncogene in retinoblastoma tumor. Our results indicate that AICAR treatment inhibited the growth of retinoblastoma tumor *in vivo* via AMPK/mTORC1 pathway and by apoptogenic, anti-proliferative, anti-angiogenesis mechanism. AICAR is a promising novel non-chemotherapeutic drug that may be effective as an adjuvant in treating Retinoblastoma.

## Introduction

Retinoblastoma is the most common primary malignant intraocular tumor in infants and children. In USA it affects 12 per million children aged 0–4 years, representing 6.1% of all childhood cancers under the age of 5 years [1]. Slightly more than half of the patients have the sporadic or non-inherited form of the disease, which results from the spontaneous inactivation of the retinoblastoma gene (RB1). In the heritable form, the patient inherits usually one defective gene from the parents and a subsequent “hit” of the uninvolved gene results in tumor formation. The heritable form is more often bilateral than the non-heritable form of the disease. Despite progress in the treatment of retinoblastoma [2], significant problems remain unsolved. Metastatic disease is often fatal [3]. Although several treatments are available for retinoblastoma, including enucleation and/or combination of chemotherapy, laser and cryotherapy, each of them has major drawbacks in pediatric patients. Conventional external beam radiation, which is used today to control large tumors, has many complications, including an increased appearance of secondary malignancies, such as osteosarcoma. This complication occurs more frequently in patients with hereditary retinoblastoma. The 30-year cumulative incidence of second malignancies is >35% for patients who received external beam therapy vs 6% for those patients without radiation [4]. Systemic chemotherapy used as a first line treatment for intraocular retinoblastoma with subsequent consolidation with photocoagulation, cryotherapy, or radiotherapy has a recurrence rate of 24% by 5 years [5]. This increases to 50% for patients with vitreous seeds [6]. Most recently analysis by the Shields and Murray groups [6], [7] show success for local control approaching 99% for RE stage I–IV, but ∼80% for RE stage V, and 90–100% for group A–C, but in less than 50% for group D (new international classification). In addition, significant morbidity with the chemotherapy has been described previously [8]. One of the drugs used for chemotherapy (etoposide) is thought to be associated with increased incidence of acute myeloblastic leukemia although the actual cases implicated so far have been low with ∼20 cases reported [9]. For these reasons, there is a need for alternative new treatment modalities for retinoblastoma with better safety and efficacy profile.

5-Aminoimidazole- 4- carboxamide riboside (AICAR) is widely used as a pharmacologic activator of AMP-activated protein kinase (AMPK). AMPK is a heterotrimeric serine/threonine protein kinase [10], which acts as a sensor of cellular energy levels and stress. Several metabolic stresses, including hypoxia, exercise, ischemia, heat shock and long-term starvation, regulate its activity [11]–[14]. Its upstream protein kinase LKB1 [15], [16] is known to be a tumor suppressor involved in Peutz-Jegher syndrome [17]. Downstream effectors of AMPK also involve the tumor suppressor Tuberous Sclerosis Complex (TSC2) and the mammalian target of Rapamycin (mTOR). Both are important known factors in cell cycle progression and tumor formation [18], [19]. AICAR is taken into cells and converted to the monophosphorylated form ZMP, mimicking an increase of AMP intracellular levels [20]. AICAR has low or no apparent toxicity and has been shown to be a great in vivo exercise mimetic [21]. Many studies have shown that AICAR can inhibit proliferation, and induce apoptosis in multiple myeloma cells [22], neuroblastoma cells [23], glioblastoma cells [24], childhood acute lymphoblastic leukemia (ALL) cells [25] colon cancer cells [26] and breast and prostate cancer cell lines [27].

We have recently demonstrated that AICAR was an efficient inhibitor of retinoblastoma cell proliferation in vitro through S-phase arrest, decrease of cyclins A and E, and partial inhibition of the mTOR pathway [28]. In the present study, we examined the in vivo effects of AICAR on Y79 Rb cell growth and demonstrated that AICAR significantly inhibited the growth of tumors in nude mice xenotransplants, by inducing apoptosis and suppressing tumor angiogenesis and macrophage infiltration.

## Materials and Methods

### Chemicals and Cell Culture

AICAR was purchased from Sigma Aldrich, St.Louis, MO, USA. AICAR was dissolved in Phosphate Buffered Saline (PBS) at concentration 67 mg/ml (260 mM) (stock solution) and stored at −20°C until utilization. The human retinoblastoma cells Y79 (ATCC, Manassas, VA, USA) were grown in RPMI medium (RPMI 1640 medium), supplemented with 20% fetal bovine serum (FBS) (Invitrogen), penicillin (100 µg/ml) –streptomycin (100 µg/ml) (Invitrogen), 2 mM l-glutamine (Invitrogen) and 10 mM HEPES (Invitrogen). Cells were incubated at 37°C in a humidified atmosphere of 95% air and 5% CO2 and split when the cells reached approximately 90% confluence.

### Animals

All animal experiments complied with the Association for Research in Vision and Ophthalmology for the use of animals in ophthalmic and vision research and were approved by the Animal Care and Use Committee of the Massachusetts Eye and Ear Infirmary (Ref #196524) (Boston, MA, USA). Four to six-week-old BALB/c (nu/nu) female mice were purchased from Charles River Laboratories (MA) and maintained in a facility under specific pathogen-free conditions. The animals were fed with pathogen free laboratory chow and allowed free access to autoclaved water in an air-conditioned room with a 12 h light/dark cycle.

### Xenograft tumor growth assay

The xenografted tumors were established by a single subcutaneous injection in each of the two flanks of 4×10^6^ Y79 retinoblastoma cells in 0.3 ml of a 1∶1 mixture of ice-cold matrigel basement membrane matrix (BD Bioscience, MA, USA) and RPMI 1640 medium supplemented with 20% FBS. Once a tumor mass became visible, three days after the injection of the cells, the mice were randomized into two groups with five mice in each group: one group receiving peritoneal injections of 500 mg/kg AICAR, the other group receiving equal volume normal saline. Mice received an injection every twenty-four hours for 28 days in total. The tumor volume was monitored by external measurement in two dimensions with calipers every other day. Tumor volume was determined according to the equation: volume (mm^3^) = 4/3×π×(length/2)×(width/2)^2^, described by Miyano-Kurosaki et al [29]. Four weeks after the first injection of AICAR, the mice were anesthetized and tumors were dissected, weighed, and stored at −80°C for further analysis.. The tumor inhibition ratio was calculated as follows: inhibition ratio (%) = [(C−T)/C]×100%, where C is the average tumor weight (or volume) of the control group and T is the average tumor weight (or volume) of the AICAR treated group. The experiment was performed on 3 independent times each time with 5 mice in each group.

### Immunohistochemistry assay and pathological evaluation

Five representative frozen tumors from each group were analyzed for retinoblastoma cell proliferation, vessel area, and macrophage infiltration. Frozen tissues were cut into 10-µm sections, fixed in 4% paraformaldehyde at room temperature for 10 min, blocked for one hour, and treated with goat serum. Tumor sections were incubated all night in a humid chamber at 4°C with primary monoclonal antibodies, including anti-Ki67 (dilution 1∶100, Dako), anti-CD31 (dilution 1∶100, BD Bioscience) and anti-CD11b (dilution 1∶100, BD Bioscience). An appropriate fluorophore-conjugated secondary antibody (Molecular Probes, Carlsbad, CA) was used to detect fluorescence using a confocal microscope (Leica Microsystems, Wetzler, Germany). Nuclei were stained with propidium iodide (PI), in the staining assay for Ki67, and with 4′, 6-diamidino-2-phenylindole (DAPI), in the staining assay for CD11b and CD31. Cryostat sections of each tumor xenograft were stained, four different fields at ×20 magnification were examined on each section and the percentage of fluorescent-positive cells/PI-positive cells or DAPI-positive cells in each field was measured. Tumor vessel area was calculated as the number of image pixels stained positive with CD31 per high-power field. In negative-control staining, the primary antibodies were omitted.

### TUNEL Analysis

To determine the degree of apoptosis, cryostat sections were prepared from tumor xenografts 31 days after implantation. Terminal dUTP nick-end labeling (TUNEL) assay was performed using the ApopTag Fluorescein In Situ Apoptosis Detection Kit (S7110, Chemicon International, Temecula, CA). Nuclei were stained with propidium iodide. The number of TUNEL (+) cells was counted in four randomly selected fields of each section from all tumor xenografts at ×20 magnification using confocal microscope.

### Protein extraction

Twelve control tumors and twelve AICAR treated tumors were chosen for analysis. The tumors were mechanically disrupted in liquid nitrogen and pieces were weighted and transferred into the pre-cooled T-PER Mammalian Protein Extraction Reagent (Thermo-Scientific, Pierce Protein Research Products) with freshly added protease (according to manufacturer suggestions; Roche Applied Science) and phosphatase inhibitor cocktails (dilution 1∶50; Thermo-Scientific, Pierce Protein Research Products). The pieces were homogenized for 15 s using rotor - stator and incubated on ice for 30 min with intermittent vortexing every 5 min. Then the samples were centrifuged for 15 min with speed 13 000 rpm in +4°C degrees. Supernatant was collected. The extraction was performed twice each time from multiple random areas of each tumor (from 12 tumors n = 24 samples analyzed).

### Western Blott Analysis

The LDS sample buffer (1∶4; Invitrogen), containing 2 microliters of 2-mercaptoethanol (Cambrex), was added to each sample. The samples were incubated at 95°C for 5 min and centrifuged briefly. Ten micrograms of total amount of proteins and thirty microliters of each sample per lane was loaded onto a 4–12% Bis-Tris Gel (NuPAGE; Invitrogen). The electrophoresis was done using NuPAGE MOPS or NuPAGE MES Running Buffer for proteins >25 kDa or <25 kDa respectively (Invitrogen) and then samples were transferred onto a PVDF membrane (0.2 micrometer; Millipore, Billerica, MA, USA). The membranes were cut and blocked for 1 h at room temperature in 5% wt/vol BSA, 1xTBS 0.05% Tween 20 at gentle shaking. The following primary anti-human monoclonal antibodies were used: p21 Waf1/Cip1 (12D1) and phospho-4E-BP1 (Ser-65) from Cell-Signaling Technology (Danvers, MA, USA), phospho-ACC (Ser-79) and phospho-S6 ribosomal protein (Ser-235/236) from Epitomics (Burlingame, CA, USA). Antihuman monoclonal GAPDH antibody from Epitomics was used as a loading control (Burlingame, CA, USA). The antibodies were diluted in 5% wt/vol BSA 1xTBS, 0.1% Tween20 as follows: p21 Waf1/Cip1 (1∶1000), phospho-4E-BP1 (1∶1000), phospho-S6 ribosomal protein (1∶20,000), p-ACC (1∶10,000) and GAPDH (1∶10,000). The blotted membranes were incubated at 4°C with gentle shaking. The following day the membranes were washed 3 times (5 min) with TBS 0.1% Tween 20 and incubated for 1 hour at room temperature with the horseradish peroxidase-labeled secondary antibodies in dilution 1∶1000 (goat antirabbit, Cell Signaling Technology, Danvers, MA, USA). The membranes were washed 3 times (5 min) in TBS 0.1% Tween 20. The immunoreactive bands were visualized with ECL or ECLprime and exposured onto Fuji RX film (Fujifilm,Tokyo, Japan). The results were quantified using ImageJ software. GAPDH was used as internal control.

### Quantitative real-time RT-PCR

Eight control tumors and eight AICAR treated tumors were chosen for qRT-PCR analysis. The tumors were mechanically disrupted in liquid nitrogen and pieces were weighted. RNA was extracted and purified with the RNeasy Midi kit (Qiagen, Valencia, CA, USA). RNA was further cleaned with an additional DNase I digestion step, according to the manufacturer's instructions. The concentration and quality of RNA was assessed using Nanodrop software and only RNA with both A260/A280 and A260/A230 >2 were selected for further analysis. Reverse transcription was performed for equal RNA amounts (4 micrograms) with OligodT primer (Invitrogen) and Superscript II (Invitrogen). cDNA 50 ng for all (except CCNE2 −100 ng, CCNA1 −200 ng) was used for each of the 4 replicates for quantitative RT-PCR. The human cyclins: A1, A2, E1, E2, D1, D2 were amplified with commercially designed exon spanning Taqman gene expression assays (Applied Biosystems, Foster City, CA, USA) and the Taqman universal PCR master mix (Applied Biosystems). GADPH, ACTB and TBP were used as independent endogenous controls. The results were acquired with a Step One Plus real-time PCR system (Applied Biosystems) and the data was calculated using comparative method described by Livek at al [30]. The extraction was performed twice each time from multiple random areas of each tumor (from 8 tumors, n = 16 samples analyzed).

### Statistical Analysis

The data are expressed as mean ± SEM (standard error of the mean). Statistical significance was evaluated using the unpaired Student's t-test and defined as P≤0.05 (*), P≤0.01 (**) or P≤0.001 (***). Two-tailed tests were used for all comparisons. The data were expressed as mean ± SEM.

## Results

### AICAR suppresses growth of human retinoblastoma xenografts

**Figure 1:**
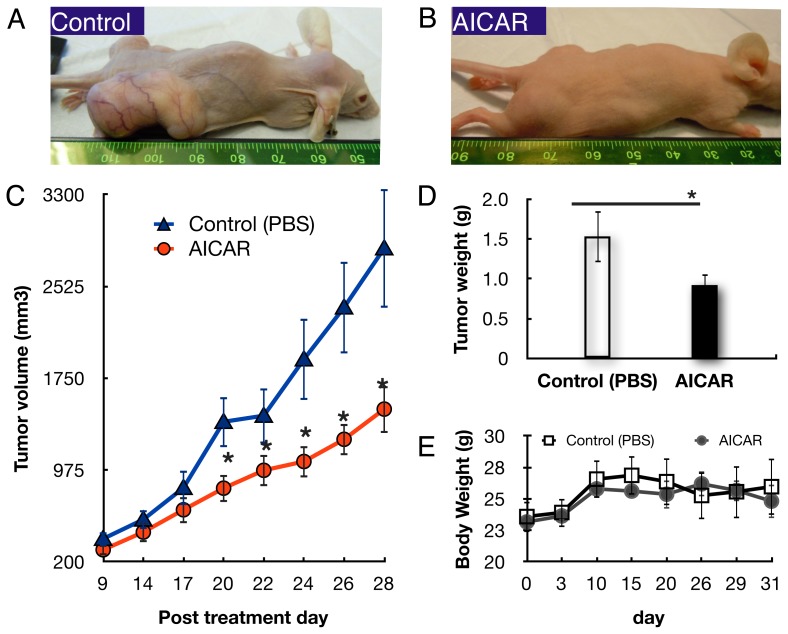
AICAR inhibited growth of xenografted tumors of Y79 human retinoblastoma cells in Nu/Nu immune-deficient mice. Human retinoblastoma Y79 cell heterotopic transplanted tumors were developed as described in Materials and Methods. Mice were treated with AICAR for 28 days. Tumor growth was monitored, and tumor tissues were collected and weighed on the 28th day after the first injection of AICAR. (A and B) Macroscopic appearance of the mice 31 days after transplantation of Y79 cells, without AICAR (A) and with 500 mg/kg/day AICAR (B). (C) Tumor growth curves: mean volumes of PBS- vs AICAR-treated group on days indicated. (D) Mean weights of tumors at autopsy of mice treated with PBS (empty column) or AICAR (filled column). (E) Effect of AICAR on body weight of mice transplanted with Y79 cells. Body weight of mice transplanted with Y79 cells with or without 500 mg/kg/day AICAR treatment was pursued for 31 days. Data are presented as mean ± SEM (n = 10).*p<0.05.

To evaluate the in vivo effect of AICAR on retinoblastoma growth, heterotopic tumor xenografts of human Y79 retinoblastoma cells were established and treated with AICAR (500 mg/kg/day, I.P.) or PBS. The appearance of the mice 28 days after treatment with or without AICAR is shown in Figure 1. In the mice treated with vehicle, large tumors were present where the Y79 cells were implanted whereas much less sizable tumors were seen in AICAR treated animals (Figure 1A,B). Compared to the PBS-treated group, AICAR suppressed tumor volume growth by 47% (p<0.03, n = 10, Figure 1C). The mean tumor weight, determined at necropsy, in the control mice was 1.53±0.32 g, as compared to 0.92±0.14 g in the AICAR-treated mice (Figure 1D) (n = 10, p<0.05). Similar results were seen if treatment was started 12 or 19 days later. The body weight of the mice was recorded bi-weekly and was not found to differ significantly among the groups (p = 0.67, Figure 1E).

### AICAR reduces human retinoblastoma Y79 cell proliferation and induces apoptosis

**Figure 2:**
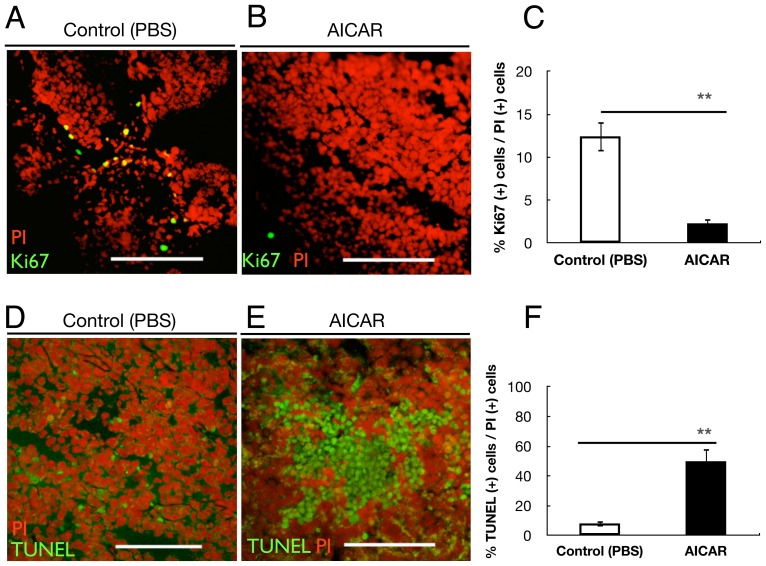
AICAR suppressed proliferation and induced apoptosis of retinoblastoma in vivo. (A, B) Immunofluorescent analysis for Ki67 of tumors of Y79 cells isolated from control mice (A) and AICAR-treated mice (B). Nuclei were stained with propidium iodide (red). (C) Quantitative analysis of Ki67 (+) cells/PI (+) cells ratio in tumors. Values are significantly lower in the AICAR-treated mice group than in the control mice group (**p<0.01). (D,E) Apoptotic cells in retinoblastoma xenografts. Typical photomicrographs of apoptotic cells using TUNEL assay (green) in Y79 xenografts. Nuclei were stained with propidium iodide (red). Y79 cells isolated from control mice (D) and AICAR-treated mice (E). (F) Quantitative analysis of the apoptotic cell percentage in tumors. Note that the number of TUNEL (+) cells was significantly higher in the AICAR-treated mice group than in the control mice group (**p<0.01). Each column represents the mean ± SEM. Scale bars (A, B, D, E), 200 µm.

To evaluate the in vivo proliferation ability of retinoblastoma cells, we examined the expression of Ki67 in four different areas from a section of five control tumors and five AICAR-treated tumors, using immunofluorescence staining. Figure 2A,B shows double staining of the cells with Ki67 and PI in the frozen sections from each tumor. The average Ki67(+)/PI(+) cells ratio was 12.44% in control mice, while it was 2.31% in AICAR-treated mice (p<0.001), showing that the proliferation ability of the cells in AICAR-treated mice was much reduced compared with the control-treated mice (Figure 2C). Apoptotic cells, with the morphology of a condensed cytoplasm and pyknotic hyperchromatic nuclei, were numerous in the sections of the tumor xenografts. To quantify the apoptotic cells in AICAR-treated and control tumors, frozen sections from each tumor were analyzed using the TUNEL assay. Figure 2E shows a typical image of the apoptotic cells with TUNEL staining in the AICAR-treated tumor. In contrast, an image of a control tumor shows significantly fewer apoptotic cells (Figure 2D). Figure 2F shows that the average number of apoptotic cells/PI(+) cells in the AICAR-treated tumors was 49.97%, as compared with 8.17% in the control tumors, representing an ∼6-fold increase (p<0.001).

### AICAR inhibits tumor angiogenesis

**Figure 3:**
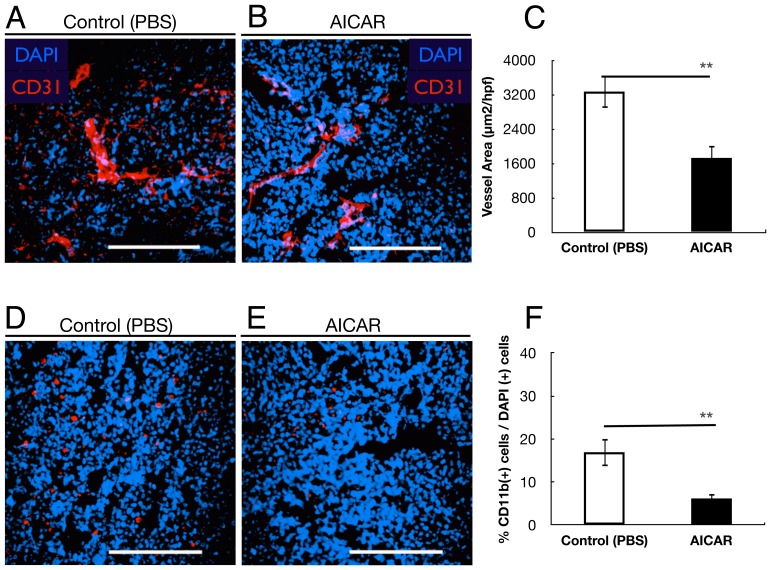
AICAR suppressed tumor angiogenesis and inflammatory cells infiltration. (A, B) Microvessel density in tumor tissues was determined by immunofluorescent staining by an endothelial-specific antibody CD31. (A) Control group and (B) AICAR-treated group. (C) Quantitative analysis of fluorescent-positive area (per 4000 µm^2^) in tumors. Vessel density was significantly suppressed in AICAR-treated mice group (**p<0.01). (D, E) Macrophage- and neutrophil- infiltration in Y79 xenografts. Typical photomicrographs of immunofluorescent staining for CD11b (red) in Y79 xenografts. Nuclei were stained with propidium iodide (blue). Y79 cells isolated from control mice (D) and AICAR-treated mice (E). (F) Quantitative analysis of the CD11b (+) cells/DAPI (+) cells ratio in tumors. The number of CD11b (+) cells was significantly lower in the AICAR-treated mice group than in the control mice group (**p<0.01). Each column represents the mean ± SEM. Scale bars (A, B, D, E), 200 µm.

The effect of AICAR on tumor angiogenesis was evaluated by CD31 immunofluorescence staining for capillaries in tumor tissues. The amount of CD31-stained tumor capillaries in the AICAR-treated group was less than in the PBS-treated group (Figure 3A,B). Morphometric analysis revealed that the microvessel density (MVD) of the AICAR-treated group was significantly reduced compared to the PBS-treated group (p = 0.003, Figure 3C). These data demonstrate that AICAR inhibits the neovascularization of retinoblastoma.

### AICAR down-regulates infiltration by CD11b(+) inflammatory cells

Inflammatory cells such as neutrophils and macrophages are thought to play an important role in tumor progression. Therefore, we analyzed the content of inflammatory cells populating tumors in the AICAR-treated group and the PBS-treated group. Interestingly, large differences were observed in the number of the CD11b(+) tumor-infiltrating neutrophils between the two groups (Figure 3D,E). Tumors isolated from AICAR-treated mice exhibited significantly lower contents of CD11b(+) cells than tumors from control mice (p = 0.002, Figure 3,D–F).

### Antiproliferative effects of AICAR are associated with activation of the AMPK pathway and inhibition of the mTORC1 pathway

**Figure 4:**
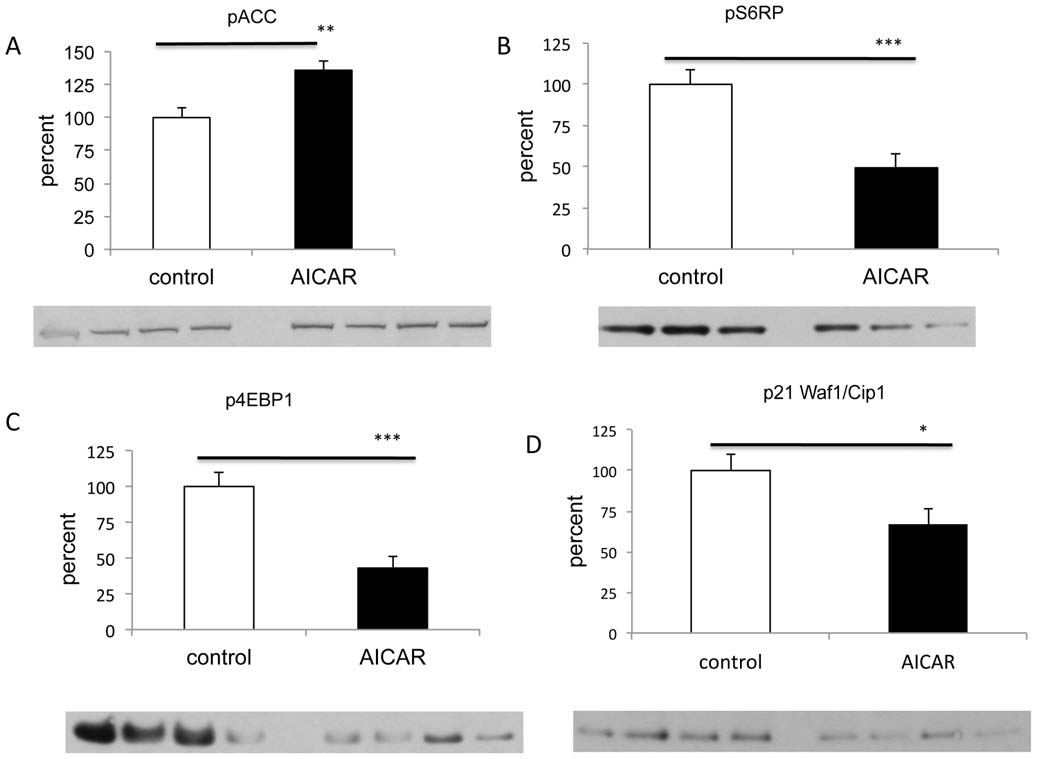
AICAR treatment of retinoblastoma is associated with activation of AMPK, inhibition of mTORC1 and decrease of p21. **A.** AICAR treatment of retinoblastoma is associated with activation of AMPK. Western blot analysis of phosphorylated ACC (Ser-79) (a downstream effector of AMPK) showed significant increase of pACC in tumours from AICAR treated mice comparing to control (**p<0.01, n = 19). **B and C.** Treatment with AICAR resulted in the inhibition of the mTORC1 pathway. Western blot analysis of tumor xenografts harvested from mice treated with AICAR showed significant decrease of mTOR pathway downstreams, pS6RP (Ser235/236) and the p4E-BP1 (Ser65) when compared to PBS-treated mice (***p<0.001 for both, n = 17 for pS6RP and n = 23 for p4EBP1). **D.** AICAR down-regulates p21WAF1/Cip1 in AICAR treated tumors as shown via Western blot analysis (*p<0.05, n = 23). Density values bands are graphically expressed relative to control. GAPDH was used as a loading control in all panels. Multiple bands represent separate biological samples. Each column represents the mean ± SEM.

To determine whether AICAR treatment in vivo was associated with AMPK activation as was observed in our in vitro study [28], we evaluated by Western blotting the phosphorylation of the immediate downstream target of AMPK, acetyl-CoA carboxylase (ACC) [31]. AICAR treated group had a 36% increase in the phosphorylation levels of ACC compared to controls (p<0.007, n = 19, Figure 4A) suggesting activation of the AMPK pathway. It has been well established that AMPK activation leads to inhibition of the mTOR pathway, resulting in dephosphorylation of ribosomal protein S6 that causes decreased initiation of translation and protein synthesis [32]–[34]. Thus we next examined the effects of AICAR on the activity of the mTOR pathway by Western blot analysis of retinoblastoma xenografts extracts. We assessed the phosphorylation status of two direct downstream targets of mTOR pathway, ribosomal S6 protein (Ser235/236) and the 4E-BP1 (Ser65) as a measure of mTOR activity. As expected, AICAR treatment was associated with reduced phosphorylation of the ribosomal S6 protein (49% vs 100%, p<0.001, n = 17, Figure 4B) and its downstream effector, 4E-BP1 when comparing to control (43% vs 100%, p<0.001, n = 23, Figure 4C). These results suggest that AICAR inhibits mTORC1 signaling in retinoblastoma in vivo mouse model.

### In vivo AICAR treatment does not affect the levels of the cyclins A, E, D in retinoblastoma, while it is associated with down-regulation of p21

**Figure 5:**
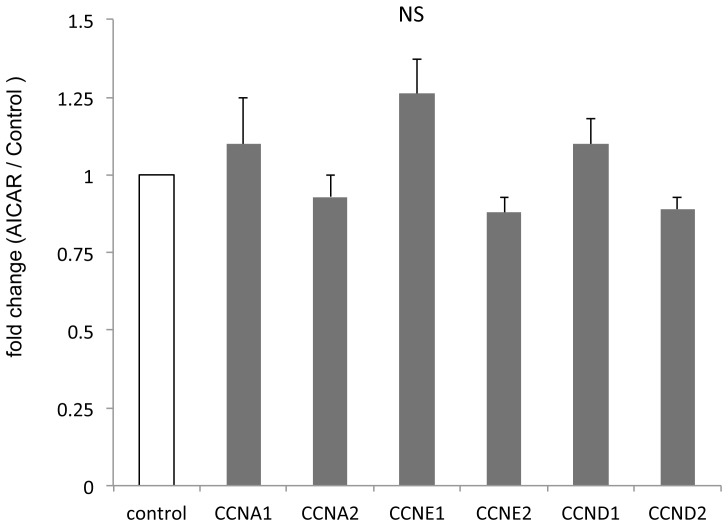
AICAR does not alter the levels of cyclins A, D and E in retinoblastoma in vivo. Quantitative RT-PCR analysis of tumors treated with AICAR in comparison with control shows no significant difference. Each column represents the mean ± SEM.

Progression of the cell cycle in eukaryotic cells is regulated by a series of serine/threonine protein kinases which consist of a catalytic subunits, cyclin dependent kinases (CDKs), and a regulatory subunits, cyclins [35]. Given the effect of AICAR on the cell cycle [28], we wanted to see whether that was mediated by changes in the levels of the appropriate cyclins. In contrast to our previous study [28], treatment with AICAR showed no change in the levels of mRNA levels of cyclins A, E, D when compared to control (n = 14; Figure 5). Interestingly, similar to our previous in vitro study [28] and in contrast to studies in other cell lines [20], [36], [37], AICAR down-regulated the protein levels of the cyclin-dependent kinase inhibitor p21 (also known as p21WAF1/Cip1) (67% vs 100%, p<0.02, n = 23; Figure 4D). Thus our in vivo and in vitro data suggest that p21 may have a unique role in regulating retinoblastoma tumor and could possibly function as an oncogene.

## Discussion

AICAR has been shown to be an exercise mimetic [21] and to have anti-cancer properties [20], [22]–[28]. The mechanisms responsible for these effects are not fully understood but they likely involve activation of AMPK. Our previous study showed that AICAR inhibits the growth of human retinoblastoma cells in vitro through inhibition of the mTOR pathway, down-regulation of cyclins A and E, and through inhibition of p21, which in retinoblastoma cells may act as an oncogene [28]. In the present study, we examined the inhibitory effects of AICAR on the growth of retinoblastoma xenografts in vivo. The growth of the retinoblastoma Y79 cells transplanted in nude mice was extensively suppressed and the size of tumor decreased to almost half of the control, after four weeks of AICAR administration (Figure 1). These results are consistent with previous reports on the in vivo anti-tumor effect of AICAR on glioblastoma, breast cancer and glioma xenografts [20], [24], [27] and suggest a potential non-chemotherapeutic strategy for retinoblastoma.

Recent studies demonstrated that AICAR inhibited cancer cells in vivo by inducing apoptosis [20] or through cytostatic mechanism [24]. We found that that the tumor mass of Y79 transplanted into nude mice treated with AICAR contained an increased number of apoptotic cells (Figure 2F) and cells with decreased mitotic figures, which may be attributed to the apoptogenic and antiproliferative activity of AICAR in vivo. A decreased Ki67 value in the masses of Y79 tumor of the mice treated with AICAR (Figure 2C) also suggested that the proliferation of the tumor was suppressed by AICAR, because Ki67 has been considered a good marker to evaluate the proliferation ability of cancers, especially of recurrent cancers [38].

Angiogenesis, the growth of new blood vessels from preexisting capillaries, is necessary for solid tumor growth and metastasis [39], [40]. Anti-angiogenesis therapy provides a novel approach for cancer management [39]. Retinoblastoma, originating from retina, maintains itself from retinal vasculature initially, and as the tumor grows and outstrips the retina, neovascularization in retinoblastoma becomes the source of tumor survival and malignant progression. Studies have shown that inhibition of the angiogenesis of retinoblastoma could be a new strategy for retinoblastoma therapy [41]. In the present study, we reported for the first time that intraperitoneal injection of AICAR inhibited retinoblastoma growth in xenografted mice and that vessel density in tumor tissues was decreased by AICAR (Figure 3A–C). In addition, AICAR suppressed macrophage infiltration (Figure 3D–F). The last result may be a reflection of less vessel infiltration or a result of less overall inflammation, as AICAR has been shown to have anti-inflammatory properties [42]–[44]

Studies have shown that VEGF is highly expressed in retinoblastoma [45] and that transfection of VEGF siRNA to retinoblastoma cells led to the inhibition of tumor growth via reduction in neovascularization [46]. In other studies AICAR and activation of AMPK has been related with cytoprotection and stimulation of angiogenesis in situations of ischemia/re-perfusion injury [47], [48]. The decrease in angiogenesis by AICAR may be an indirect effect of the decreased tumor mass rather than a direct effect on angiogenesis. AMPK stimulating angiogenesis under ischemia condition [47], [48] may be related to its protective effect on endothelial cells in stress. The inhibition of angiogenesis in cancer may be attributed to its effects on production and secretion of cytokines. Recently, Zhou et al [49] reported that AMPK upregulates TNFSF15, a cytokine that exerts a potent inhibitory effect on tumor angiogenesis. It is, also, possible that the various effects of AICAR depend on the specific cell type, cellular events following external stimuli, paracrine effects and/or downstream-regulated pathways.

Proliferation of cancer cells requires oncogenic growth signals as well as sufficient metabolic energy for biogenesis of cellular constituents [50]. The “Warburg effect” [51], a metabolic derangement in cancer cells resulting in increased glucose uptake and glycolysis, provides a selective advantage to rapidly proliferating tumor cells by supplying cellular bioenergetics required to support tumor progression. Cells must coordinate diverse processes including cell division, cell migration, and cell polarity with the cell's metabolic status. AMPK is posited to function as a central sensor/regulator of energy status within the cell, and could thus have direct roles in linking metabolism to cell division [34]. It can interface with diverse signaling molecules ranging from LKB1 to mammalian target of rapamycin [34]. The mTORC1 is directly inhibited by phosphorylation of raptor as a consequence of activation of the AMPK [52]. The mTOR kinase pathway regulate translation repressor protein (4EBP1) activity *in vivo* [53], [54] via phosphorylation of various 4E-BP1 residues [55]. When hypophosphorylated, the 4EBP1 binds tightly to eIF4E, preventing proper formation of the eIF4 translation initiation complex at the 5 end of cap-bearing mRNAs [56], [57]. Hyperphosphorylation of 4E-BP1 at Ser65 disrupts this interaction thus eIF4E is released, allowing it to associate with eIF4G and other relevant factors to promote cap-dependent translation [56], [58]. In our in vivo study, we showed that AICAR treatment induced the activation of AMPK, and inhibited mTOR signaling indicated by dephosphorylation of pS6RP (Ser235/236) and p4EBP-1 (Ser65) in retinoblastoma tumor xenografts (Figure 5 A,B,D). Decreased AMPK activation has been found in some cancers [59], [60] and mTOR signaling is has been activated many tumors [61], which may become an attractive target for cancer therapy.

Progression of the cell cycle in eukaryotic cells is regulated by a series of serine/threonine protein kinases which consist of a catalytic subunit, cyclin dependent kinases (CDKs), and a regulatory subunits, cyclins [35]. Whereas in our in vitro study we observed changes in the mRNA levels of cyclins A, E and D after AICAR administration, we did not observe any significant differences after in vivo administration. In contrast our in vitro findings of down regulation of p21 was also observed in the in vivo study. Cdk-interacting protein 1 (Cip1 or p21) is a 21-kDa protein known as inhibitor of cell cycle progression and tumor suppressor, owing to its ability to inhibit the activity of CDK–cyclin complexes and proliferating cell nuclear antigen (PCNA) [62]–[64]. Both our in vitro and in vivo studies have seen a paradoxical down-regulation of p21 in AICAR inhibited retinoblastoma. This paradoxical down-regulation of p21 has not been reported in any previous study of AICAR effects on cancer cells. Two possible explanations are that either p21 was down-regulated as a compensatory mechanism, or p21 acts as an oncogene in retinoblastoma cells. Interestingly, p21 has been shown to be overexpressed in a variety of human cancers including prostate, cervical, breast and squamous cell carcinomas and, in many cases, p21 upregulation correlates positively with tumor grade, invasiveness and aggressiveness and is a poor prognostic indicator [37]. Some recent studies suggest that, under certain conditions and in some tumors, p21 family can promote cellular proliferation, act as a positive regulator of the cell cycle and inhibit apoptosis [37], [65], [66]. Interestingly, the studies of Gartel and Radhakrishnan [67] suggest that p21 may act as a positive regulator of the cell cycle. In fact, mitogenic stimuli result in transient p21 induction during G1-S progression. Thus, when p21 is repressed in such a context, it will lead to impairment of cell cycle progression due to decreased complex formation of cyclin D-cdk4/cdk6. This may be one of the mechanisms of AICAR inhibition of Rb cells and their arrest in S phase. Together, these data suggest that depending on the cell environment, p21 may function as either a tumor suppressor or an oncogene and both our studies suggest that p21 may have a novel function as an oncogene in human retinoblastoma tumor.

**Figure 6:**
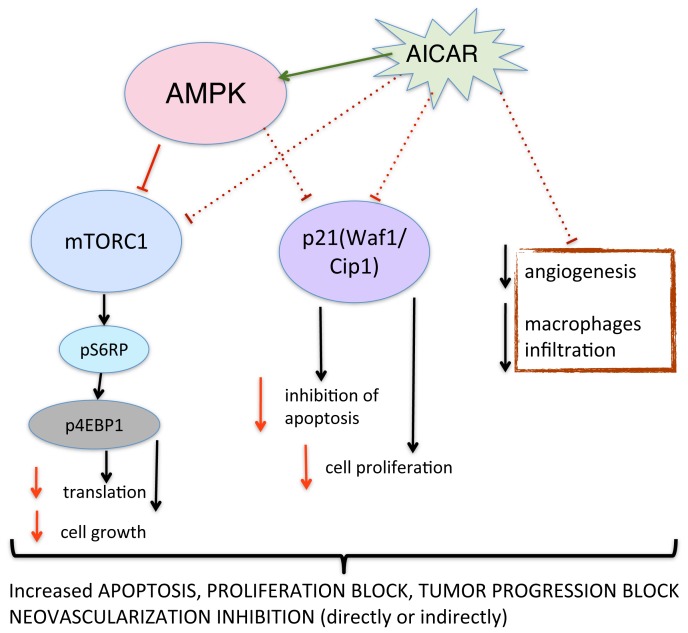
Proposed mechanism of action for AICAR in human retinoblastoma in an in-vivo xenograft model. AICAR administration leads to activation of AMPK decreased tumor vessel density and decreased CD11b (macrophage) infiltration. Activated AMPK inhibits mTOR pathway, protein synthesis and cell growth. In addition, AICAR administration results in decreased levels of p21, which was recently found to inhibit apoptosis and promote cell proliferation. Overall signaling changes leads to loss of viability due to apoptosis, proliferation block and inhibition of tumor progression.

Our study demonstrates that AICAR significantly suppresses the growth of retinoblastoma in vivo by apoptogenic and anti-proliferative activity and is associated with decreased angiogenesis and inhibition of macrophage infiltration (Fig. 6). We replicated in vivo our in vitro finding of paradoxical down-regulation of p21 in retinoblastoma after AICAR administration, which indicates that p21 may have a novel function of an oncogene in retinoblastoma tumor. The studies of AICAR's anti-inflammatory properties [43], [44], exercise mimetic features [21], and anti-proliferative effects in vitro and in vivo, provide a foundation for future clinical strategies that utilize AICAR and AMPK activation by AICAR or any other pharmacological agent as an attractive target for cancer therapy as a single agent or in combination with other first-line agents to improve treatment.
